# The effectiveness of peripheral compartment first access and periportal capsulotomy technique for arthroscopic management of femoroacetabular impingement: A prospective case series

**DOI:** 10.5152/j.aott.2021.21174

**Published:** 2021-11-01

**Authors:** Emre Anıl Özbek, Mehmet Yağız Ayduğan, Ramazan Akmeşe

**Affiliations:** 1 Department of Orthopedics and Traumatology, İbn’i Sina Training and Research Hospital, Ankara University, Ankara, Turkey; 2 Department of Orthopaedic Surgery, Haliç University, School of Medicine, İstanbul, Turkey

**Keywords:** Capsular thinning, FAI, Hip arthroscopy, Peripheral compartment first access, Periportal capsulotomy

## Abstract

**Objective:**

The aim of this study was to evaluate the functional results of hip arthroscopy for femoroacetabular impingement (FAI) performed via the periportal capsulotomy technique combined with capsular thinning and peripheral compartment first access.

**Methods:**

This prospective study included 34 patients (20 female, 14 male; mean age = 32.3 ± 12.5 years) treated for combined type FAI and labral tears between January 2016 and January 2018. In radiographic evaluation, center-edge angle (CEA) and alpha angle were measured preoperatively and postoperatively. Patients’ functional status was assessed at 3, 6, 12, and 24 months using the modified Harris Hip Score (mHHS), Hip Disability and Osteoarthritis Outcome Score - Activities of Daily Living (HOOS-ADL), and Hip Disability and Osteoarthritis Outcome Score - Sports-Specific Subscale (HOOS-SSS), and visual analog scale (VAS).

**Results:**

The mean alpha angle decreased from 55.5°±2.9° preoperatively to 48.3° ± 2.6° postoperatively. The mean CEA decreased from 39.2° ± 3.0° preoperatively to 32.9° ± 2.6° postoperatively. The mean duration of surgery was 96.7 ± 21.1 minutes; the mean traction time was 45.5 ± 14.6 minutes. The mean mHHS at the 3^rd^ , 6^th^, 12^th^, and 24^th^ months showed a statistically significant increase compared to the preoperative value (*P* < 0.05). The mean HOOS-ADL and HOOS-SSS at the postoperative 3^rd^, 6^th^, and 12^th^ months demonstrated a statistically significant increase compared to the preoperative values (*P* < 0.05). The same scores measured at the 24th month, however, did not demonstrate a significant increase. The mean VAS scores at the 3^rd^ and 6^th^ months postoperative illustrated a significant decrease compared to the preoperative values (*P* < 0.05) whereas this significant decrease was not observed at the 12^th^ and 24^th^ months.

**Conclusion:**

The combined technique of periportal capsulotomy and capsular thinning used in this study seems to be a reliable surgical method with favorable functional results, a low complication rate, and a low risk of hip instability.

**Level of Evidence:**

Level IV, Therapeutic Study

## Introduction

With the increasing indications in the last decade, Hip Arthroscopy (HA) is now most often used in cases with Femoroacetabular Impingement (FAI). Although the ligaments forming the hip joint capsule play a key role in joint stability, they constitute an obstruction to visualization in HA surgery and prevent the comfortable use of instruments by surgeons.^1,2^ Good and excellent functional results have been reported for HA used in the treatment of FAI.^3^ Although there are technical difficulties, such as visualization of the Peripheral Compartment (PC) and Central Compartment (CC), these can be overcome with capsulotomy.^4^

For visualization of the PC during HA, several surgeons use the interportal capsulotomy method in which the iliofemoral ligament is cut from the Anterolateral Portal (ALP) as far as the mid-anterior or direct Anterior Portal (AP).^5^ Some surgeons prefer having a T-shaped capsulotomy by adding an incision extending distally in addition to the interportal capsulotomy.^6^ There are studies in the literature that have reported that the hip joint capsule plays a key role in joint stability and that unrepaired capsulotomies can lead to poor clinical results in the long term through postoperative microinstability.^5,7^ On the other hand, some researchers have reported that, in terms of postoperative functional results, a repaired capsulotomy is not superior to an unrepaired or partially repaired capsulotomy.^8^ The limited periportal capsulotomy technique, which does not require capsule repair, was described by Chambers et al.^6,9^ The authors described their technique with the central access first method, as a full capsulotomy was not made, a working area could be created within the PC by moving the working instruments and the camera in the same direction, and this was reported to be necessary to ensure that there was no remaining residual impingement with fluoroscopy.^9^

One of the most common postoperative complications of HA is neurological deficit; and one of the main causes of a neurological deficit has been reported to be the traction method, used especially in visualization of the CC.^1,10^ Dienst et al. described the peripheral access first method to reduce traction-related complications, by shortening the traction time.^11^ To facilitate instrumentation use with visualization inside the PC and avoid wide capsulotomy during this method, capsular thinning has been described.^12^ With direct visualization and no requirement for fluoroscopy, entry to the CC can be provided with this method and a lower risk of iatrogenic labrum and chondral injury is undertaken.^1,13^

The purpose of this study was to evaluate the functional results of HA performed for the treatment of FAI using the periportal capsulotomy technique combined with capsular thinning and PC first access. The hypothesis of the study was that the periportal limited capsulotomy technique combined with the PC first access would provide good or excellent postoperative functional results.

## Materials and Methods

Approval for this prospective study was granted by the Ethics Committee at the School of Medicine of Ankara University (I5-272-20). Informed consent was obtained from all patients in the study. Hip arthroscopy surgery was performed on 77 patients with pincer, cam, or mixed types of FAI at our clinic between January 2016 and January 2018. The study included 34 (20 females, 14 males) of the 77 patients who had a combined type impingement and labral tears.

The study inclusion criteria were defined as persistent hip pain not responsive to conservative treatment for 3 months, clinical findings consistent with FAI (pain with flexion-internal rotation and adduction), a labral tear determined with MRI, an alpha angle > 55°(on MRI), and a Center-Edge Angle (CEA) > 40°. The study exclusion criteria were defined as a history of HA, ipsilateral hip fracture, revision hip AS, osteoarthritis (Tönnis Grade 2-3), hip dysplasia (CEA < 25°), and hip hypermobility (Beighton score ≥ 4).^14^

The demographic data of the patients including age, gender, and Body Mass Index (BMI) were recorded. Preoperative and postoperative pelvis AP, 45°Dunn and false profile X-ray views, and preoperative MRIs were taken. Total duration of surgery, traction time, and intraoperative and postoperative complications were also noted.

### Surgical technique

**Figure 1.**
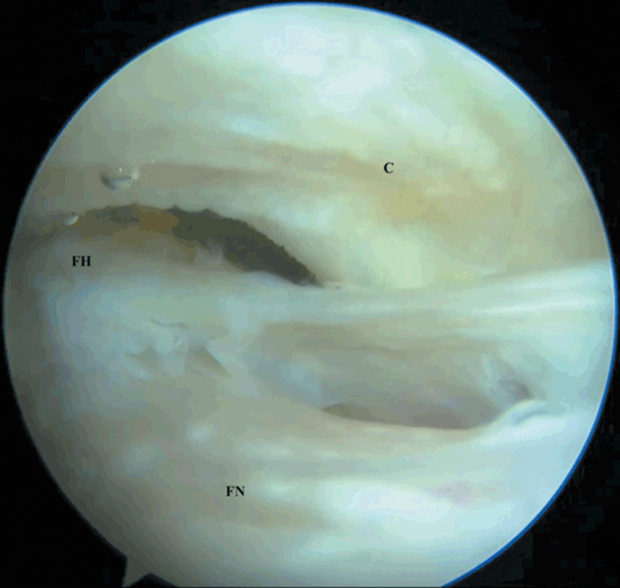
Hip arthroscopy in a patient with FAI in the right hip. The 70°arthroscope is placed via the anterolateral portal. Peripheral compartment is presented without hip traction. C: hip capsule, FH: femoral head, and FN: femoral neck.

All surgeries were performed by the same orthopedic sports medicine surgeon who had more than 10 years of experience in HA surgery. Patients were administered hypotensive general anesthesia (systolic blood pressure < 90 mmHg) and laid in the supine position on a traction table (Advanced Supine Hip Positioning System; Smith + Nephew, Inc., Andover, MA, USA). For the peripheral access first method, the joint was reached by opening a Proximal Anterolateral Portal (PALP) as the “visualization portal” under fluoroscopic guidance with the hip positioned in 40° to 50° of flexion.^11^ Then, as the “working portal”, an AP was created.^11^ The capsule was widened 8-10 mm around the portals, and the periportal capsulotomy was performed ensuring that the capsulotomy incisions did not join under 70°arthroscopy control (Figure 1).^15^

**Figure 2.**
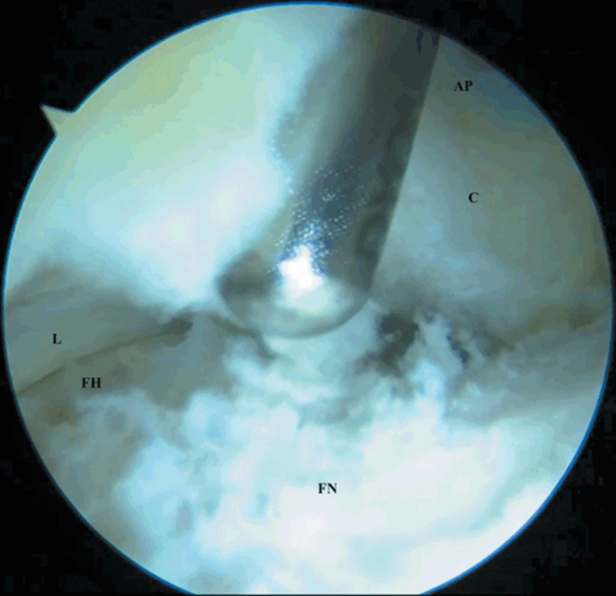
Hip arthroscopy in a patient with FAI in the right hip. The 70°arthroscope is placed via the anterolateral portal and a 4.5-mm shaver is placed via the anterior portal. Capsular thinning is performed with the shaver. AP: Anterior Portal, C: Hip Capsule, FH: Femoral Head, FN: Femoral Neck, and (L) Labrum.

**Figure 3.**
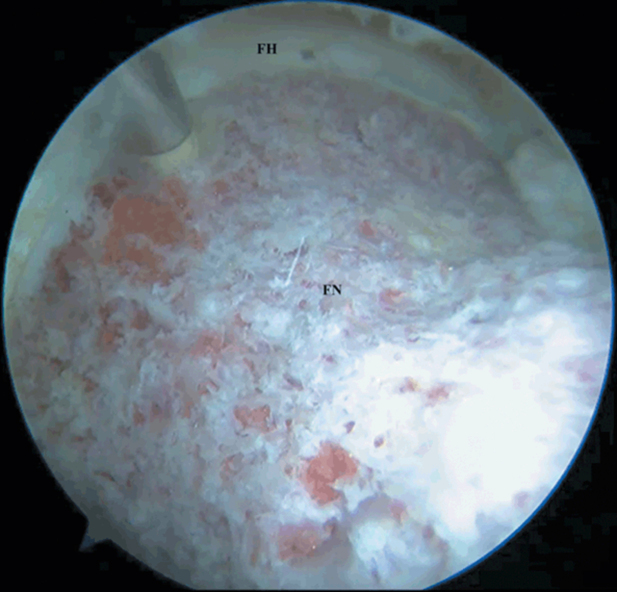
Hip arthroscopy in a patient with FAI in the right hip. The 70°arthroscope is placed via the anterolateral portal and radiofrequency ablation device is placed via the anterior portal. Capsular thinning and femoroplasty were performed. A wide working area in the peripheral compartment without hip traction can also be seen. FH: Femoral Head and FN: Femoral Neck.

**Figure 4.**
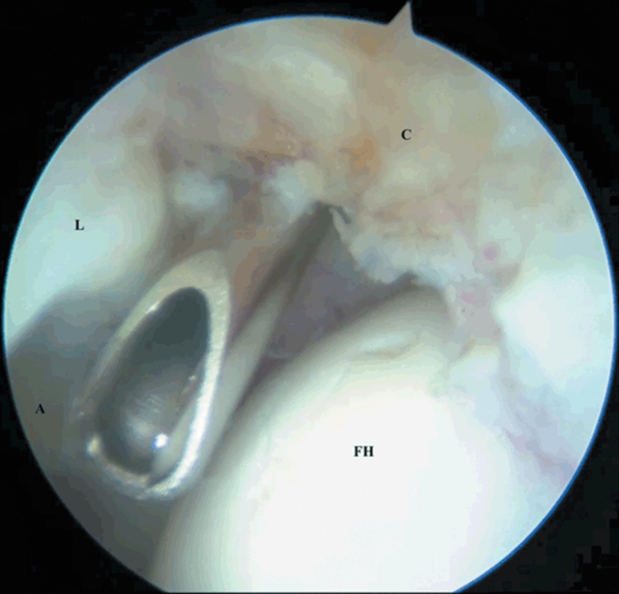
Hip arthroscopy in a patient with FAI in the right hip. The 70°arthroscope is placed via the anterolateral portal. A 4.5-mm shaver is also placed via the anterior portal in the central compartment with hip traction. A: acetabulum, C: hip capsule, FH: femoral head, and L: labrum.

Capsular thinning was applied by thinning the zona orbicularis and surrounding capsule from within the joint with a shaver (DYONICS POWERMAX; Smith + Nephew, Inc.) and a radiofrequency ablation (RFA) device (Arthrocare; Smith + Nephew, Inc.) (Figure 2).^16^ Femoroplasty was performed with a 5.5-mm burr (DYONICS POWERMAX; Smith + Nephew, Inc.) under fluoroscopic guidance with the hip positioned in 40°-50°of flexion. While continuing the femoroplasty procedure, a wider working area was formed by the arthroscopic fluid pressure creating a balloon effect in the capsule (Figure 3).^12^ To check whether the femoroplasty was sufficient, the hip was checked with fluoroscopy 0°, 30°, and 60°externally rotated while in flexion, then the femoroplasty procedure was terminated. Traction was applied by taking the hip into extension in order to pass to the CC. Under arthroscopic guidance, the CC was entered through an ALP. Using the ALP as the imaging portal in the CC, the AP was used as the working portal (Figure 4).^11^ Just as in the PC, the periportal capsulotomy was applied for this portal under 70° arthroscopy control. After completing the standard diagnostic arthroscopy procedure in the CC, pincer FAI was revealed and under fluoroscopic guidance, acetabuloplasty was performed with a 5.5-mm burr. Since all patients in this study were treated with labrum repair, no labrum debridement was performed in any patient.

The labrum repair was made using 1.8 mm suture anchors (Q-Fix All-Suture Anchor; Smith + Nephew, Inc.) and the number of anchors to be used was decided intraoperatively according to the size of the labral tear. The capsule was checked again with a 70°arthroscope, and when it was ensured that the capsulotomy incisions had not expanded, the traction was terminated.

Postoperatively, the patients were given crutches for mobilization for the first 6 weeks. After one week of touch-down weight-bearing, a gradual increase in weight-bearing with the help of physical therapy was allowed to reach full weight-bearing in the 6^th^ week.

The Tönnis grade, CEA, and alpha angles were measured from the preoperative and postoperative radiological images and recorded. The patient-reported outcomes (PROs) included the modified Harris Hip Score (mHHS), Hip Disability and Osteoarthritis Outcome Score - Activities of Daily Living (HOOS-ADL), Hip Disability and Osteoarthritis Outcome Score - Sports-Specific Subscale (HOOS-SSS), and visual analog scale (VAS) score. These PROs were recorded preoperatively, and at the postoperative 3^rd^, 6^th^, 12^th^, and 24^th^ months.

### Statistical analysis

Data obtained in the study were analyzed using the SPSS for Windows v.20 software (IBM SPSS Corp.; Armonk, NY, USA). Continuous data including age, BMI, pre- and postoperative alpha angles, CEA, and PROs were compared with the paired and unpaired Student’s t-tests and Tukey’s post-hoc analysis. Categorical data, including the side of surgery and gender were compared with a Chi-square test. A value of *P* < 0.05 was considered statistically significant.

## Results

The patients had a mean age of 32.3 ± 12.5 years and a mean BMI of 24.9 ± 4.2. Surgery was applied to the right hip in 23 (68%) patients and the left hip in 11 (32%). The mean alpha angle was measured as 55.5° ± 2.9° preoperatively and 48.3° ± 2.6° postoperatively. The mean CEA was measured as 39.2° ± 3.0° preoperatively and 32.9° ± 2.6° postoperatively. The mean duration of surgery was 96.7 ± 21.1 min, while the mean traction time was 45.5 ± 14.6 min (Table 1). None of the patients required revision HA or Total Hip Arthroplasty (THA).

The mHHS, HOOS-ADL, HOOS-SSS, and VAS scores were measured and recorded as PROs preoperatively and at the 3^rd^, 6^th^, 12^th^, and 24^th^ month postoperatively (Table 2). The data of each scoring system were compared at the different time points during the follow-up period.

The mean mHHS at the 3^rd^, 6^th^, 12^th^, and 24^th^ months showed a statistically significant increase compared to the preoperative value (*P* < 0.05). The mean HOOS-ADL and HOOS-SSS at the postoperative 3^rd^, 6^th^, and 12^th^ months showed a statistically significant increase compared to the preoperative values (*P* < 0.05). The same scores measured at the 24^th^ month, however, did not demonstrate a significant increase compared to the 12^th^ month measurements (Table 2). A statistically significant decrease was determined in the mean VAS pain score at the 3^rd^ and 6^th^ months postoperative compared to the preoperative values (*P* < 0.05). However, this significant decrease was not observed at the 12^th^ and 24^th^ months.

## Discussion

The results of this study demonstrated that satisfactory results were obtained in patients treated with acetabuloplasty, femoroplasty, and labral repair with the periportal capsulotomy technique combined with capsular thinning and PC first access HA method. In our search, we found no other study in the literature that included all patients operated on with acetabuloplasty, femoroplasty, and labral repair with the periportal capsulotomy method.

Methods, such as interportal capsulotomy and T-capsulotomy, have been described to obtain better visualization in the PC during HA.^5^ However, the subject of capsular management in HA surgery remains a matter of debate because of the hip instability that can develop as a result of cutting close to the iliofemoral ligament in all these capsulotomy methods.^5,17^ In the 5-year follow-up results of a study where the capsule was left closed or not closed, Domb et al. reported that there was no difference with respect to revision HA (15.4%), and that higher PRO scores and a lower requirement for hip arthroplasty surgery could be obtained with capsule closure (18.5% and 10.8%, respectively).^18^ In another study, Frank et al. compared full capsule closure to partial capsule closure over a 2.5-year follow-up period and reported that similar PRO results were obtained in both groups, while there was a higher rate of requirement for revision HA in the group where the capsule was not closed (13% and 0%, respectively).^19^ In addition to these results, there are also studies in the literature reporting that there could be loss of range of motion in the hip joint with capsule closure.^20^ In a prospective, a 2-year follow-up study by Economopoulos et al.,^5^ the comparison of T-capsulotomy without capsule repair and interportal capsulotomy with and without capsule repair were compared. The results showed no difference between the groups with respect to requirement for revision HA, whereas the need for THA was lowest (8%, 0%, and 0%, respectively) and the results of PROs were highest in the group where the capsule was closed.

The results of another study by Chambers et al. with a 2-year follow-up showed that in patients who underwent periportal capsulotomy with no need for capsule closure, no hip instability was observed postoperatively, and a statistically significant increase was reported in the postoperative PRO results compared to the preoperative values.^6^ In our study, visualization of the PC was obtained with the combination of periportal capsulotomy and capsular thinning. As a result of this prospective study, there was no requirement for revision HA or THA in any patient, and satisfactory PROs were obtained similar to those of other methods, such as capsule closure and periportal capsulotomy recommended in the literature.

Hip arthroscopy is a reliable surgical method for FAI, which is an early etiology for hip osteoarthritis, resulting in satisfactory patient outcomes postoperatively.^21,22^ Therefore, the short- and mid-term outcomes of HA have been the subject of several studies in the literature. Domb et al. reported that better results were obtained with capsular repair, and that in their 5-year follow-up, the mean mHHS was 80.8 ± 16.1, the mean HOOS-Sports was 68.1 ± 27.4, and the mean VAS score was 2.5 ± 2.4.^18^ Frank et al. reported that better functional results could be obtained with complete capsular repair compared to partial capsular repair during their 2-year follow-up period, and found a mean mHHS of 83 ± 4.8, mean HOOS-ADL of 92 ± 7.9, and a mean HOOS-Sport of 87.3 ± 8.2 in their study.^19^ In a study by Moon et al. in which capsule management methods were not defined, a group with a 2 year follow-up, treated with combined type FAI and labral repair, had a mean mHHS of 93.6 (range: 63-100).^21^ In another study by Chambers et al.,^6^ HA was performed on 142 patients with periportal capsulotomy using a technique similar to that of the current study, and the 2-year follow-up results were reported retrospectively. No statistically significant increase was reported in the 2-year follow-up values compared to the 1-year values, with mean mHHS reported as 88.5 ± 15.9, mean HOOS-ADL as 87.9 ± 19.0, mean HOOS-Sports as 75.5 ± 2.6, and mean VAS score as 1.8 ± 2.5. Similar to our study, no statistically significant improvement was determined in the VAS values after 6 months, or in the HOOS-ADL and HOOS-Sports values after 1 year. At the end of the two year period, the PRO values returned a mean mHHS of 87.1 ± 5.8, mean HOOS-ADL of 88.3 ± 4.5, mean HOOS-Sports of 76.1 ± 4.5, and mean VAS score of 1.3 ± 1.0. These results can be considered to show satisfactory results similar to those of other capsular management methods recommended in the literature.

As HA is being performed more often by orthopedic surgeons, there are studies in the literature reporting the trend towards labral repair rather than labral debridement during surgery.^23^ There are also studies stating that the learning curve for HA is steep and that satisfactory patient results can be obtained after the first 20 surgeries, while the operating times decrease after the first 30 surgeries.^24,25^ In a study by Dumont et al. which reported the results of HA with capsule repair performed by a single surgeon, the first 75 cases were operated on in 143.7 ± 29.3 min, while in the second 75 cases, the operating time significantly decreased to a mean of 102.8 ± 24.3 min.^26^ Benedetto et al.^27^ included cam and/or pincer FAI patients in a study where all patients underwent labral repair but no capsule repair was performed. In their study, the mean duration of surgery was reported to be 76 (range: 56-103) min. In a study by Chambers et al., which included patients with cam and/or pincer FAI patients and/or operated on with labral repair with periportal capsulotomy with no requirement for capsule repair, the mean duration of surgery was 93.5 ± 23.2 min and the mean traction time was 56.5 ± 17.6 min.^6^ The patients in our study were treated with acetabuloplasty, femoroplasty, and labral repair, all performed by a single arthroscopy surgeon with more than 10 years of experience. Again in the current study, the mean operating time was 96.7 ± 21.1 min and the traction time was 45.5 ± 14.6 min, which were close to the results of other studies which included a heterogeneous patient group.^13,28^ When the procedures performed in the operation are considered, the operating times in the current study can be considered to be almost the same as the other operating times reported in the literature. This capsular thinning provides ease of working in the PC, thus the periportal capsulotomy did not require capsule closure.

Although satisfactory results were obtained with the technique we used, there were some limitations to our study. The most important limitation was the relatively low number of patients, which was due to forming a homogenous patient group in which acetabuloplasty, femoroplasty, and labral repair were performed together. This study is a case series of one surgeon’s experience, which could be deemed as another limitation. Finally, the absence of a control group and the fact that a follow-up period of two years may be considered relatively short are other limitations.

In conclusion, HA is a method increasingly applied by orthopedic surgeons, which provides satisfactory postoperative results. The combined technique of periportal capsulotomy and capsular thinning used in this study seems to be a reliable method with a low complication rate and a low risk of hip instability. Furthermore, it can be considered that while satisfactory PRO results can be obtained with this method with no requirement for capsule closure, the operating time can be shortened as the experience of the surgeon increases.

**Highlights**

- The combined technique of periportal capsulotomy and capsular thinning used in this study seems to be a reliable method with a low complication rate and a low risk of hip instability.
- Satisfactory patient-reported outcome results can be obtained with this method with no requirement for capsule closure.
- With direct visualization and no requirement for fluoroscopy, entry to the central compartment can be provided with this method and a lower risk of iatrogenic labrum and chondral injury is undertaken.

## Figures and Tables

**Table 1.** Patients demographics.

Age (years)	32,3 ± 12.5
Women (n, %)	20 (58%)
BMI	24.9 ± 4.2
Right side involved (n, %)	23 (67%)
Alpha angle (preop.- postop.)	55.5 (±2.9)-48.3 (±2.6)
CEA (preop.- postop.)	39.2 (±3)-32.9 (±2.6)
Procedure time (min)	96.,7 ± 21.1
Traction time (min)	45.5 ± 14.6

BMI, Body Mass Index; CEA, Center-Edge Angle.

**Table 2.** Mean patient-reported outcome (PRO) scores.

PROs/follow-up	Properative	3 month	6 month	1 year	2 year
VAS pain score	6.68 ± 1.39	3.53 ± 0.99	1.82 ± 0.67	1.12 ± 0.98	1.38 ± 1.01
mHHS	54.62 ± 5.06	65.06 ± 4.44	72.26 ± 2.99	77.38 ± 2.92	87.18 ± 5.88
HOOS – ADL	42.82 ± 9.54	63.94 ± 2.83	73.74 ± 5.75	85.79 ± 4.97	88.35 ± 4.56
HOOS – sports	51.91 ± 7.87	62.79 ± 6.24	67.18 ± 5.57	73.79 ± 5.85	76.18 ± 4.52

HOOS-ADL, Hip Disability and Osteoarthritis Outcome Score - Activities of Daily Living; HOOS-Sports, Hip Disability and Osteoarthritis Outcome Score - Sports; mHHS, Modified Harris Hip Score; VAS Pain Score, Visual Analog Pain Score.

## References

[1] Marin-Peñai O. Basic concepts in hip arthroscopy. In: Gmmj K Haddad F Hirschmann M Karlsson J Seil R , editors. ESSKA Instructional Course Lecture Book. Berlin, Heidelberg: Springer; 2018.45-67.

[2] Ng KCG Jeffers JRT Beaulé PE. Hip joint capsular anatomy, mechanics, and surgical management. J Bone Joint Surg Am. 2019;101(23):2141-2151. 10.2106/JBJS.19.00346 31800428 PMC7406151

[3] Acuña AJ Samuel LT Roth A Emara AK Kamath AF. How capsular management strategies impact outcomes: A systematic review and meta-analysis of comparative studies. J Orthop. 2020;19:237-243. 10.1016/j.jor.2020.02.002 32071521 PMC7016034

[4] Gédouin J-E. Arthroscopic treatment of femoroacetabular impingement: Technical review. Orthop Traumatol Surg Res. 2012;98(5):583-596. 10.1016/j.otsr.2012.06.001 22795065

[5] Economopoulos KJ Chhabra A Kweon C. Prospective randomized comparison of capsular management techniques during hip arthroscopy. Am J Sports Med. 2020;48(2):395-402. 10.1177/0363546519894301 31891553

[6] Chambers CC Monroe EJ Flores SE Borak KR Zhang AL. periportal capsulotomy: technique and outcomes for a limited capsulotomy during hip arthroscopy. Arthrosc J Arthrosc Relat Surg Off Publ Arthrosc Assoc North Am Int Arthrosc Assoc. 2019;35(4):1120-1127. 10.1016/j.arthro.2018.10.142 30871902

[7] Wuerz TH Song SH Grzybowski JS, . Capsulotomy size affects hip joint kinematic stability. Arthrosc J Arthrosc Relat Surg Off Publ Arthrosc Assoc North Am Int Arthrosc Assoc. 2016;32(8):1571-1580. 10.1016/j.arthro.2016.01.049 27212048

[8] Domb BG Stake CE Finley ZJ Chen T Giordano BD. Influence of capsular repair versus unrepaired capsulotomy on 2-year clinical outcomes after arthroscopic hip preservation surgery. Arthrosc J Arthrosc Relat Surg Off Publ Arthrosc Assoc North Am Int Arthrosc Assoc. 2015;31(4):643-650. 10.1016/j.arthro.2014.10.014 25530511

[9] Monroe EJ Chambers CC Zhang AL. Periportal capsulotomy: A technique for limited violation of the hip capsule during arthroscopy for femoroacetabular impingement. Arthrosc Tech. 2019;8(2):e205-e208. 10.1016/j.eats.2018.10.015 30906690 PMC6411513

[10] Kocaoğlu H Başarır K Akmeşe R, . The effect of traction force and hip abduction angle on pudendal nerve compression in hip arthroscopy: A cadaveric model. Arthrosc J Arthrosc Relat Surg Off Publ Arthrosc Assoc North Am Int Arthrosc Assoc. 2015;31(10):1974-80. 10.1016/j.arthro.2015.03.040 26033463

[11] Dienst M Seil R Kohn DM. Safe arthroscopic access to the central compartment of the hip. Arthrosc J Arthrosc Relat Surg Off Publ Arthrosc Assoc North Am Int Arthrosc Assoc. 2005;21(12):1510-1514. 10.1016/j.arthro.2005.09.014 16376244

[12] Apivatgaroon A Dienst M. Compression and flip test for diagnosis of unstable acetabular labral tears using a peripheral compartment approach. Arthrosc Tech. 2016;5(6):e1433-e1439. 10.1016/j.eats.2016.08.014 28560140 PMC5439082

[13] Dantas P Gonçalves S Mascarenhas V Camporese A Marin-Peña O. Hip arthroscopy with initial access to the peripheral compartment provides significant improvement in FAI patients. Knee Surgery, Sport Traumatol Arthrosc. 2021;29(5):1453-1460. 10.1007/s00167-020-06380-z 33386879

[14] Naal FD Hatzung G Müller A Impellizzeri F Leunig M. Validation of a self-reported Beighton score to assess hypermobility in patients with femoroacetabular impingement. Int Orthop. 2014;38(11):2245-2250. 10.1007/s00264-014-2424-9 24993650

[15] Tang H-C Brockwell J Dienst M. Hip arthroscopy via a peripheral compartment first capsular-preserving technique: A step-by-stepdescription. J Hip Preserv Surg. 2020;00(0):1-8. 10.1093/jhps/hnaa061 PMC8081412 33948216

[16] Dantas P Gonçalves S Mascarenhas V Barreira M Marin-Peña O. Hip arthroscopy with initial access to the peripheral compartment: A detailed step-by-step technique description. Arthrosc Tech. 2020;9(11):e1651-e1655. 10.1016/j.eats.2020.07.006 33294322 PMC7695549

[17] Strickland CD Kraeutler MJ Brick MJ, . MRI evaluation of repaired versus unrepaired interportal capsulotomy in simultaneous bilateral hip arthroscopy: A double-blind, randomized controlled trial. J Bone Joint Surg Am. 2018;100(2):91-98. 10.2106/JBJS.17.00365 29342058

[18] Domb BG Chaharbakhshi EO Perets I Walsh JP Yuen LC Ashberg LJ. Patient-reported outcomes of capsular repair versus capsulotomy in patients undergoing hip arthroscopy: Minimum 5-year follow-up-a matched comparison study. Arthrosc J Arthrosc Relat Surg Off Publ Arthrosc Assoc North Am Int Arthrosc Assoc. 2018;34(3):853-863. 10.1016/j.arthro.2017.10.019 29373289

[19] Frank RM Lee S Bush-Joseph CA Kelly BT Salata MJ Nho SJ. Improved outcomes after hip arthroscopic surgery in patients undergoing T-capsulotomy with complete repair versus partial repair for femoroacetabular impingement: A comparative matched-pair analysis. Am J Sports Med. 2014;42(11):2634-2642. 10.1177/0363546514548017 25214529

[20] Domb BG Stake CE Lindner D El-Bitar Y Jackson TJ. Arthroscopic capsular plication and labral preservation in borderline hip dysplasia: Two-year clinical outcomes of a surgical approach to a challenging problem. Am J Sports Med. 2013;41(11):2591-2598. 10.1177/0363546513499154 23956133

[21] Moon J-K Yoon JY Kim C-H Lee S Kekatpure AL Yoon PW. Hip arthroscopy for femoroacetabular impingement and concomitant labral tears: A minimum 2-year follow-up study. Arthrosc J Arthrosc Relat Surg Off Publ Arthrosc Assoc North Am Int Arthrosc Assoc. 2020;36(8):2186-2194. 10.1016/j.arthro.2020.04.041 32389770

[22] Lindman I Öhlin A Desai N, . Five-year outcomes after arthroscopic surgery for femoroacetabular impingement syndrome in elite athletes. Am J Sports Med. 2020;48(6):1416-1422. 10.1177/0363546520908840 32195598 PMC7227125

[23] Westermann RW Day MA Duchman KR Glass NA Lynch TS Rosneck JT. Trends in hip arthroscopic labral repair: An American board of orthopaedic surgery database study. Arthrosc J Arthrosc Relat Surg Off Publ Arthrosc Assoc North Am Int Arthrosc Assoc. 2019;35(5):1413-1419. 10.1016/j.arthro.2018.11.016 30979629

[24] Konan S Rhee S-J Haddad FS. Hip arthroscopy: Analysis of a single surgeon’s learning experience. J Bone Joint Surg Am. 2011;93(Suppl 2):52-56. 10.2106/JBJS.J.01587 21543689

[25] Lee YK Ha YC Hwang DS Koo K-H. Learning curve of basic hip arthroscopy technique: CUSUM analysis. Knee Surg Sports Traumatol Arthrosc. 2013;21(8):1940-1944. 10.1007/s00167-012-2241-x 23073816

[26] Dumont GD Cohn RM Gross MM Menge TJ Battle NC Thier ZT. The learning curve in hip arthroscopy: Effect on surgical times in a single-surgeon cohort. Arthrosc J Arthrosc Relat Surg Off Publ Arthrosc Assoc North Am Int Arthrosc Assoc. 2020;36(5):1293-1298. 10.1016/j.arthro.2019.11.121 31805387

[27] Di Benedetto P Gorasso G Castriotta L Mancuso F Giardini P Causero A. All-suture anchors in arthroscopic acetabular labral repair: Our experience. Acta Biomed. 2020;91(1):85-91. 10.23750/abm.v91i4-S.9661 PMC7944822 32555081

[28] Rupp R Duggan B. Peripheral versus central compartment starting point in hip arthroscopy for femoroacetabular impingement. Orthopedics. 2012;35(2):e148-53. 10.3928/01477447-20120123-02 22310398

